# The limiting effect of genome size on xylem vessel diameter is shifted by environmental pressures in seed plants

**DOI:** 10.1002/pld3.471

**Published:** 2022-12-14

**Authors:** Xiangyan Feng, Linfei Zhong, Hai Zhou, Jingwen Bi, Huma Batool, Xintan Zhang, Wenzhi Zhao

**Affiliations:** ^1^ Linze Inland River Basin Research Station, Key Laboratory of Ecohydrology of Inland River Basin, Northwest Institute of Eco‐Environment and Resources Chinese Academy of Sciences Lanzhou China; ^2^ University of Chinese Academy of Sciences Beijing China; ^3^ College of Geography and Environment Science Northwest Normal University Lanzhou China; ^4^ School of Life Sciences Fudan University Shanghai China; ^5^ Sardar Bahadur Khan Women's University Quetta Pakistan; ^6^ College of Agriculture Nanjing Agricultural University Nanjing China

**Keywords:** cell volume, environmental pressure, genome size, plasticity, vessel diameter

## Abstract

Current and previous studies have extensively studied the physiological and ecological consequences of genome size (GS) on plants because of the limiting effect of GS on cell size. However, it is still obscure whether such limiting effect could be shifted by environmental pressures, or not. Here, we compiled a global dataset comprised of GS, xylem vessel diameter (*V*_dia_), xylem hydraulic conductivity (*K*_S_), *P*_50_ (xylem water potential at the loss of 50% maximum *K*_S_), and climate factors of 251 phylogeny and habitat divergent species from 59 families. The results showed that GS could limit the *V*_dia_ of the species from the same family sampled in the similar climate conditions. But the expected positive relationship between GS and *V*_dia_ became uncertain and even negative across different environmental conditions. *V*_dia_ was strongly positively coordinated with mean annual temperature (MAT), mean annual precipitation (MAP), and potential evapotranspiration (PET). Furthermore, *V*_dia_ as the anatomic foundation of plant hydraulic performance was strongly positively coordinated with *K*_S_ and negatively coordinated with −*P*_50_. The strong environmental selection on *K*_S_ and *P*_50_ explained the concerted regulation of *V*_dia_ by environmental factors. The findings revealed the combined regulation of GS and environmental pressures on xylem cell size and thus affected plant eco‐physiological performance. The shifted cell size limiting effect of GS by environmental factors manifests plants great plasticity under changed environmental conditions.

## 1 INTRODUCTION

Genome size (GS) is a key plant trait shaping plant physiological performance and ecology strategy (Pandit et al., 2014). The main perspective proposed that GS is positively coordinated with cell size (Commoner, 1964; Price et al., 1973), thus influencing the plant functional traits such as seed size (Knight et al., 2005; Thompson, 1990) and guard cell size (Beaulieu et al., 2008). Because of the cell size limiting effect by GS, the ecological and physiological consequences of GS have attracted great attention and were thought as the indicator of plant ecological strategy. For instance, genome downsizing has been proposed as the prerequisite for the success of angiosperms because small GS allows for easier construction of dense minute stomata cells and promotes efficient gas exchange compared with gymnosperms (Simonin & Roddy, 2018). In addition, species with relatively small GS are thought as the advantage of species invasiveness because of the invasive species with small GS with fast growth rate and resource acquisition rate compared with native species with large GS (Pandit et al., 2014; te Beest et al., 2012).

Similar to angiosperm genome downsizing, the evolution of advanced vascular system has also been considered an indicator of plant success through the deep time (Brodribb, Carriqui, et al., 2020). Among the vascular and hydraulic traits, *K*_S_ and *P*_50_ are the two dominant traits shaping species ecological performance, survival, and drought resistance across different climates and lineages (McDowell et al., 2019; Nardini & Luglio, 2014; Scoffoni et al., 2016). In specific, *K*_S_ is quantified by the magnitude of xylem‐specific hydraulic conductivity. Great *K*_S_ is achieved by great vessel diameter (*V*_dia_) because the water transport capacity scales with vessel diameter to the fourth power (Ooeda et al., 2017). Under water deficit conditions, the failure of the hydraulic transport system could directly result in plant mortality (Brodribb, Powers, et al., 2020). The index used to evaluate plant vulnerability to hydraulic failure is called hydraulic safety and is quantified by *P*_50_; the water potential at half of the maximum hydraulic conductivity is lost, and the more negative value represents, the greater hydraulic resistance to water deficit. Typically, great *V*_dia_ coordinated with less −*P*_50_ (low safety) because of the high risk to embolism of wider vessels, vice versa (He et al., 2020; Liu et al., 2019; Santiago et al., 2018).

**FIGURE 1:**
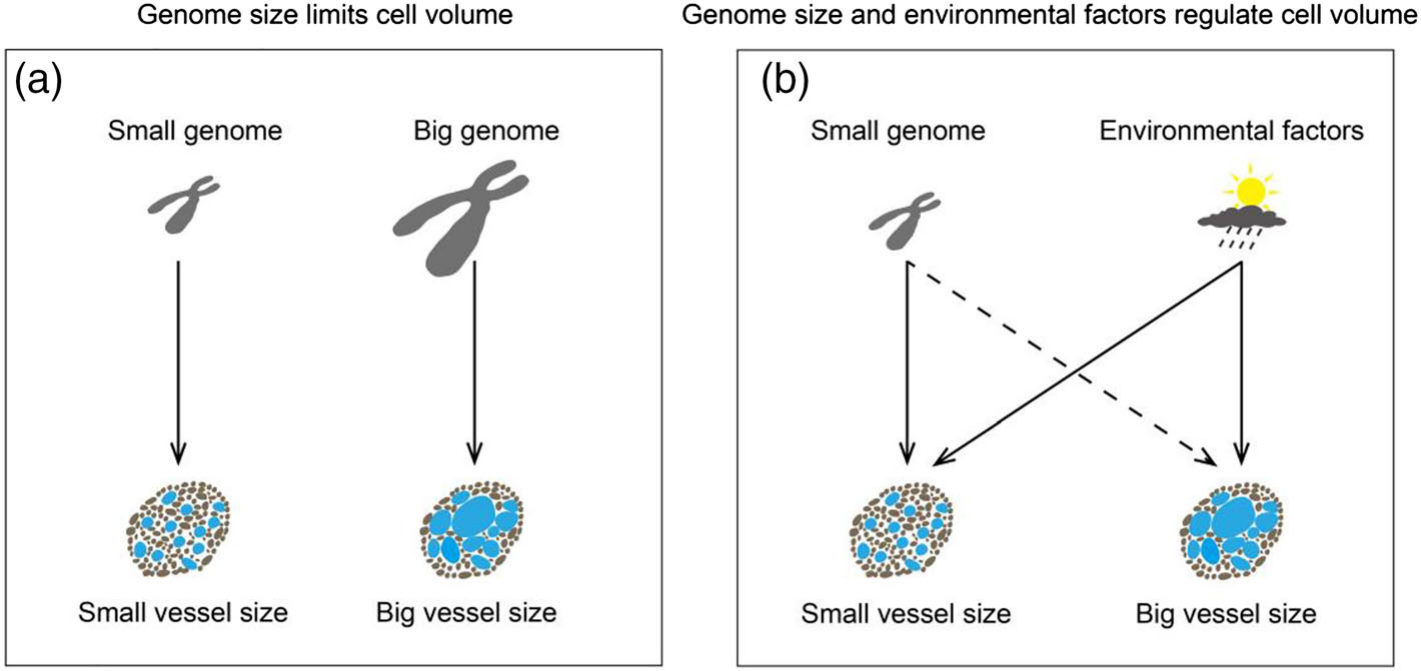
The conceptual model of genome size and environmental factors regulate xylem vessel size. (a) According to the genome size limiting cell volume, small genome size coordinated with small vessel size, vice versa. (b) The relationship between genome size and vessel volume becomes uncertain under the changed climate conditions. In such condition, the environmental factors would shift the limiting effect of genome size on vessel size.

According to the GS limiting cell size theory, *V*_dia_ should be limited by GS as well as the other cell types, that is, small GS is expected to be coordinated with small *V*_dia_ and large GS coordinated with big *V*_dia_ (Figure 1a). Besides the limiting effect of GS on *V*_dia_, environmental pressures are also the strong regulators on *V*_dia_. For environmental factors, precipitation and temperature are the key environmental pressures for *K*_S_ and *P*_50_. In the region with low precipitation and transpiration demand, low *K*_S_ and high −*P*_50_ (low hydraulic efficiency and high hydraulic safety) are expected for species to acclimate to the environments with insufficient water supply and high embolism risk. Because *V*_dia_ is the fundamental anatomic structure of *K*_S_ and *P*_50_, thereby, small *V*_dia_ should be evolved in such environments. In contrast, in the moist and warm regions, species would evolve great *K*_S_ and low −*P*_50_ (high hydraulic efficiency and low hydraulic safety) to cope with adequate water availability and high transpiration demand, which is corresponding to great *V*_dia_ (He et al., 2020). Combining the regulating roles of GS and environmental factors on *V*_dia_, we hypothesized that the species with small GS habituated in the environment would favor low *K*_S_ and high −*P*_50_, because of species with both small GS and small *V*_dia_; for the species with small GS habituated in the moist environments favored great *K*_S_ and low −*P*_50_, the limiting effect of GS on *V*_dia_ would possibly be shifted by environmental pressures (Figure 1b).

To test the hypothesis that the limiting effect of GS on *V*_dia_ might be shifted by environmental pressures, we compiled a global dataset of 251 divergent seed plants comprised by GS, *V*_dia_, *K*_S_, mean annual temperature (MAT), mean annual precipitation (MAP), and potential evapotranspiration (PET). First, to prove the limiting effect of GS on *V*_dia_ in the similar climate conditions, we analyzed the relationship between GS and *V*_dia_ of the species sampled from the same sites in six families including both angiosperms and gymnosperms. Second, to explore whether such limiting effect of GS on *V*_dia_ could be shifted by environmental pressures, we analyzed the relationship between GS and *V*_dia_ of the species sampled across different sampling sites. Finally, we analyzed the relationships between environmental factors and *V*_dia_ by analyzing the relationships of *V*_dia_ with *V*_dia_, *K*_S_, and environmental factors. We hypothesized that the limiting effect of GS on *V*_dia_ exists in the species distributed in the similar environmental conditions and the relationship between GS and *V*_dia_ could be shifted across different environmental conditions; the fine regulation of environmental pressures on *V*_dia_ could be explained by the environmental selection on *K*_S_ and *P*_50_.

## 2 MATERIALS AND METHODS

### 2.1 Data compilation

We compiled a global dataset comprised of 251 species of globally distributed seed plants from 59 families (Table S1). The compiled dataset includes GS, xylem vessel diameter (*V*_dia_), xylem hydraulic conductivity (*K*_S_), *P*_50_ (xylem water potential at the loss of 50% maximum *K*_S_), MAT, MAP, and PET. GS information of all studied species was extracted from the Plant DNA C‐values database (https://cvalues.science.kew.org/) (Pellicer & Leitch, 2020), which is an open‐accessed genome dataset constructed by Royal Botanic Gardens, Kew. In order to assure the data matching of the dataset, we directly extracted *V*_dia_, *K*_S_, *P*_50_, MAT, MAP, and PET from a high‐quality dataset recently published by Liu et al. (2019). In the newly compiled dataset, GS, traits data, and environmental factors were well matched.

### 2.2 Data analysis

We first analyzed whether the limiting effect of GS on *V*_dia_ could be shifted by environmental pressures. Because of the great GS differences of the species from different families, we analyzed the relationships between GS and *V*_dia_ of the species from the same family in the similar and across different environmental conditions. To exclude the environmental effects on the relationship between GS and *V*_dia_, we analyzed the relationship between GS and *V*_dia_ of the species of six families representing angiosperms and gymnosperms sampled at the same sampling sites. To illustrate whether environmental pressures could shift the limiting effect of GS on *V*_dia_, we analyzed the relationships between GS and *V*_dia_ across different sampling sites. Furthermore, to further prove the uncertain relationships of GS and *V*_dia_ across different environmental conditions, the overall relationships between GS and *V*_dia_ of another species from eight families were analyzed. Simple linear model function *lm* in R programming was used to get the overall change trends of *V*_dia_ along GS of the species from different families in the same and across sites.

Second, we analyzed how the environmental pressures regulating *V*_dia_. The relationships of *V*_dia_ with MAT, MAP, and PET were analyzed for both angiosperms and gymnosperms. Because *V*_dia_ is the fundamental anatomic foundation of *K*_S_ and *P*_50_, we further analyzed the relationships of *V*_dia_ with *K*_S_ and *P*_50_. The coordination relationships between environmental factors and *K*_S_ and *P*_50_ were analyzed as well. To homogenize variance, all the continuous variables were naturally log‐transformed. The coordination among variables was analyzed by standardized major axis analysis (SMA) by smatr package in R (Warton et al., 2012).

## 3 RESULTS

### 3.1 The limiting effect of GS on *V*_dia_ was shifted by environmental pressures

**FIGURE 2:**
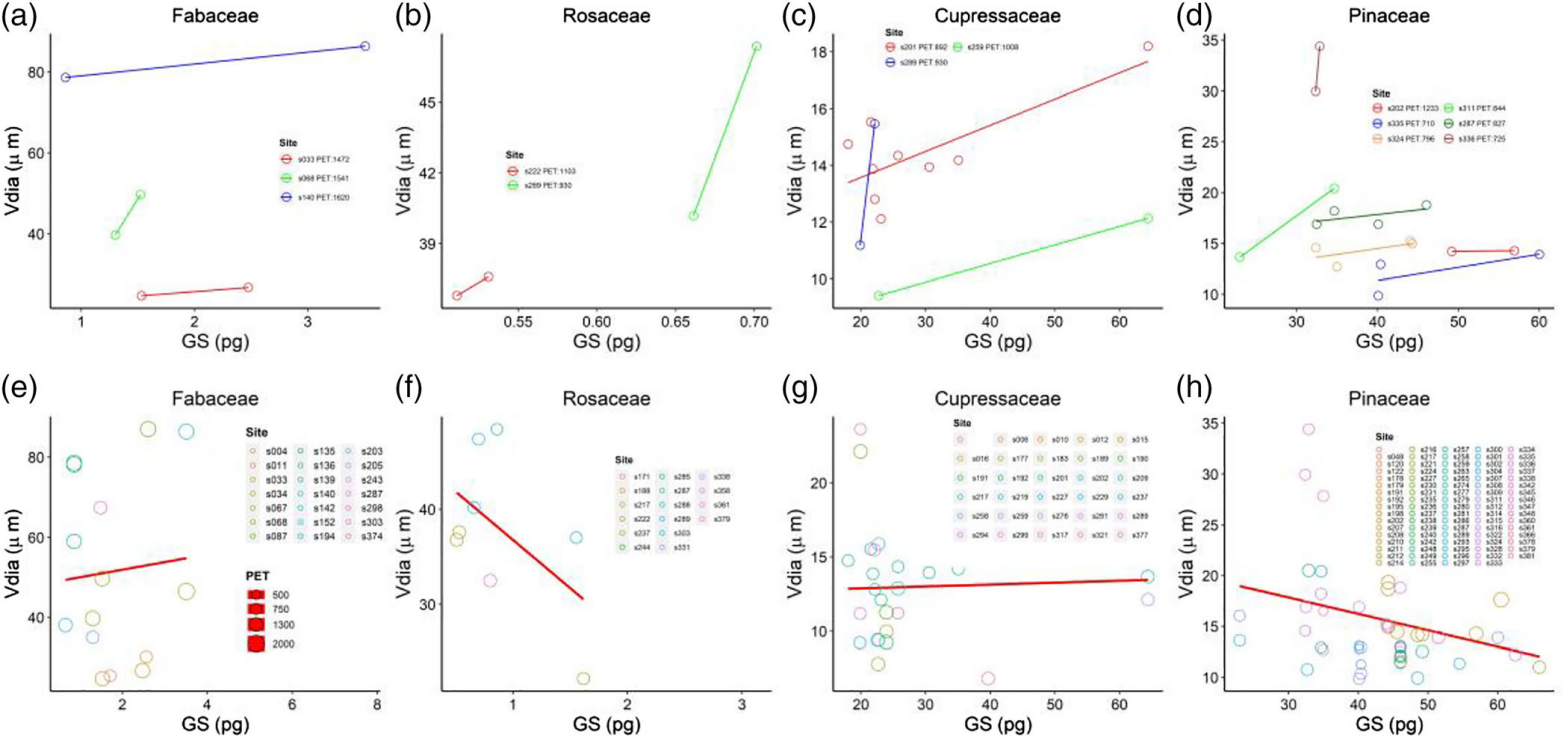
The limiting effect of genome size on vessel diameter was shifted by environmental factors. (a), (b), (c), (d) In the same sampling sites, the relationship between GS and *V*_dia_ is positive in the four families (*n* = 6, *n* = 4, *n* = 13, *n* = 16, respectively). (e), (f), (g), (h) The relationship between GS and *V*_dia_ became uncertain when analyzed across sites with different environmental conditions in the same families (*n* = 21, *n* = 16, *n* = 30, *n* = 64, respectively). (e), (g) The relationships keep positive or (f), (h) changed negatively. Each circle represents one species. The circles with the same color represent the species in the family sampled from the same site. In (a), (e), (b), and (f), the site number and potential evapotranspiration are shown in labels. In (c), (d), (g), and (h), the potential evapotranspiration is represented by circle size. Different colors represent different sampling sites as the legend showed. The colored circle without site information are labeled by blank space in (g) and (h). GS, genome size; *V*_dia_, vessel diameter, PET, potential evapotranspiration. (a), (e), (b), and (f) represent two angiosperm families; (c), (d), (g), and (h) represent two gymnosperm families.

**FIGURE 3:**
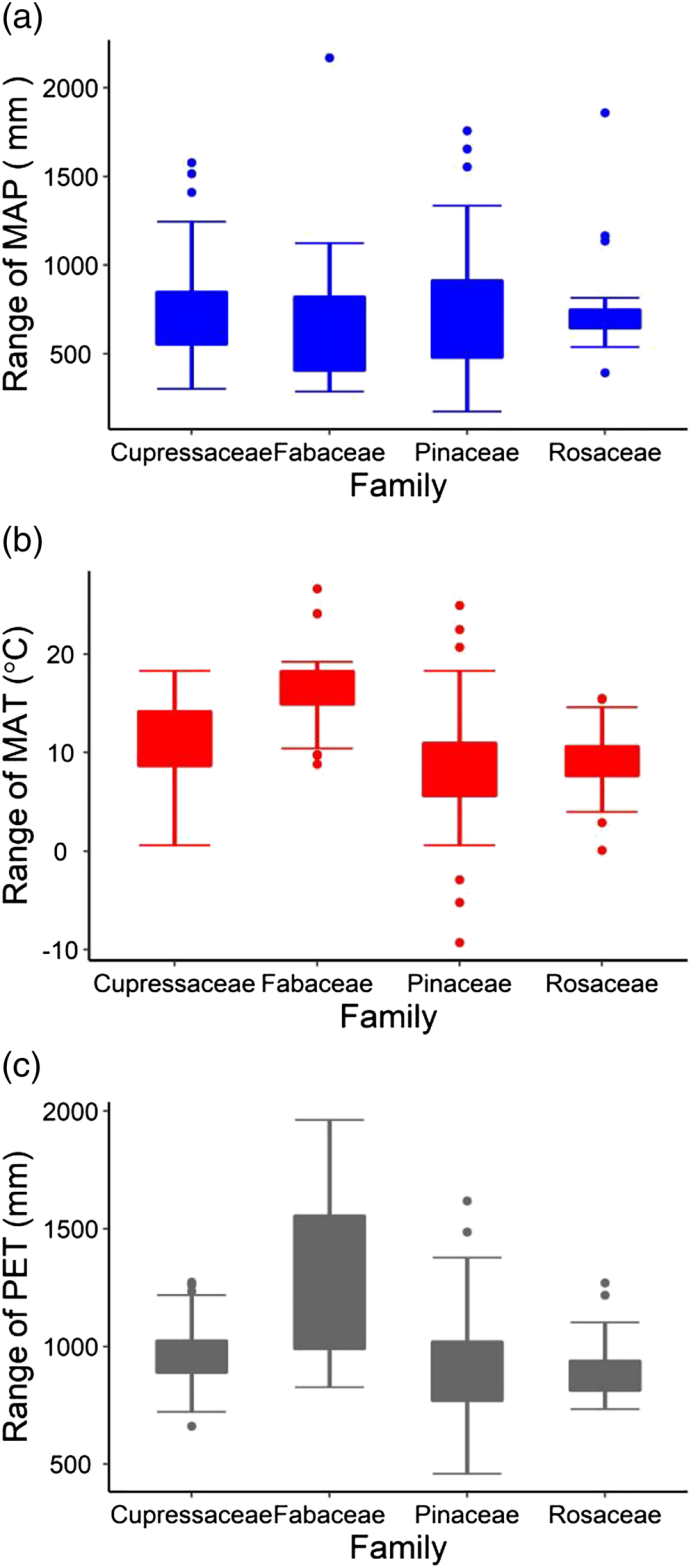
The environmental pressure that the species from four families experienced. Boxplot of the habitat (a) MAP, (b) MAT, and (c) PET range that Cupressaceae, Fabaceae, Pinaceae, and Rosaceae experienced. MAT ranged from 100 to 2000 mm, MAT ranged from 0 to 20°C, and PET ranged from 500 to 2000 mm. MAT, mean annual temperature; MAP, mean annual precipitation; PET, potential evapotranspiration

The result showed that, in the same sampling sites, the positive relationship between GS and *V*_dia_ of the species from same family was found. Specifically, we found the positive relationships between GS and *V*_dia_ in 16 sites among six families (Figures 2a–d and S1a,b). It verified that, under the same environmental condition, the limiting effect of GS on *V*_dia_ is rather clear as well as the other cell types (von Sachs, 1893, (Beaulieu et al., 2008). To figure out whether the limiting effect of GS on *V*_dia_ could be shifted by environmental factors, we analyzed the relationship between GS and *V*_dia_ across different sampling sites. The result showed that the relationship between GS and *V*_dia_ became uncertain when the analysis was conducted across environments (Figures 2e–h and S1c,d). As the expected limiting effect of GS on *V*_dia_, the negative relationship between GS and *V*_dia_ should not be observed. However, the positive relationships were detected only in three families (Figures 2e,g and S1c), and the negative relationships were detected in another three families (Figures 2f,h and S1d). It means that the limiting effect of GS on *V*_dia_ could be shifted by environmental factors, in which, the relationship between GS and *V*_dia_ still keeps positive or shifted negatively. Consistent with the hypothesis, species from four families all experienced strong environmental pressure (Figure 3). In specific, MAT ranged from 100 to 2000 mm, MAT ranged from 0 to 20°C, and PET ranged from 500 to 2000 mm (Figure 3). We further analyzed the relationship between GS and *V*_dia_ of the species from another additional eight families across different environmental conditions. The uncertain relationships between GS and *V*_dia_ were shown as well (Figure S2). The positive relationships between GS and *V*_dia_ were only found in three families (Figure S2b,d,f), and the negative relationships were found in another five families (Figure S2a,c,e,g,h).

### 3.2 *V*_dia_ was finely tuned by environmental factors attributed to the environmental selection on hydraulic performance (*K*_S_ and *P*_50_)

**FIGURE 4:**
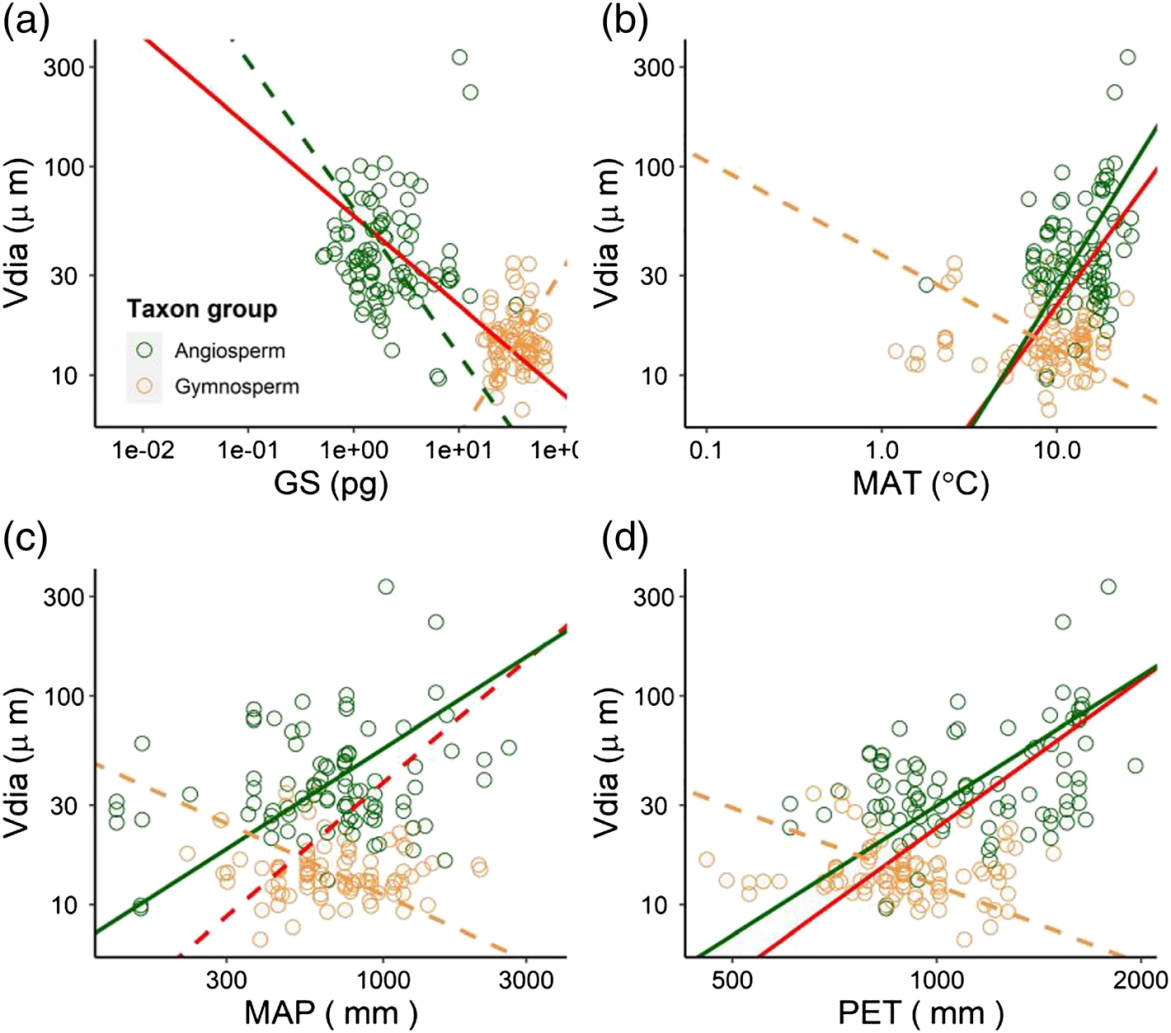
The relationships of vessel diameter with genome size and environmental factors in different taxon. (a) GS is negatively coordinated with *V*_dia_, negatively in angiosperms, and positively in gymnosperms (*n* = 185). (b) MAT is positively coordinated with *V*_dia_, positively in angiosperms, and negatively in gymnosperms (*n* = 178). (c) MAP is positively coordinated with *V*_dia_, positively in angiosperms, and negatively in gymnosperms (*n* = 178). (d) PET is positively coordinated with *V*_dia_, positively in angiosperms, and negatively in gymnosperms (*n* = 178). GS, genome size; *V*_dia_, vessel diameter; MAT, mean annual temperature; MAP, mean annual precipitation; PET, potential evapotranspiration. Green and yellow circles represent angiosperms and gymnosperms, respectively. Red lines represent the curved line that pooled all angiosperms and gymnosperms points. Solid curved lines represent the relationship at the significant level (*P* < .05), and dashed curved lines represent the relationship as insignificant (*P* > .05). The model parameters are reported in Table S2.

**FIGURE 5:**
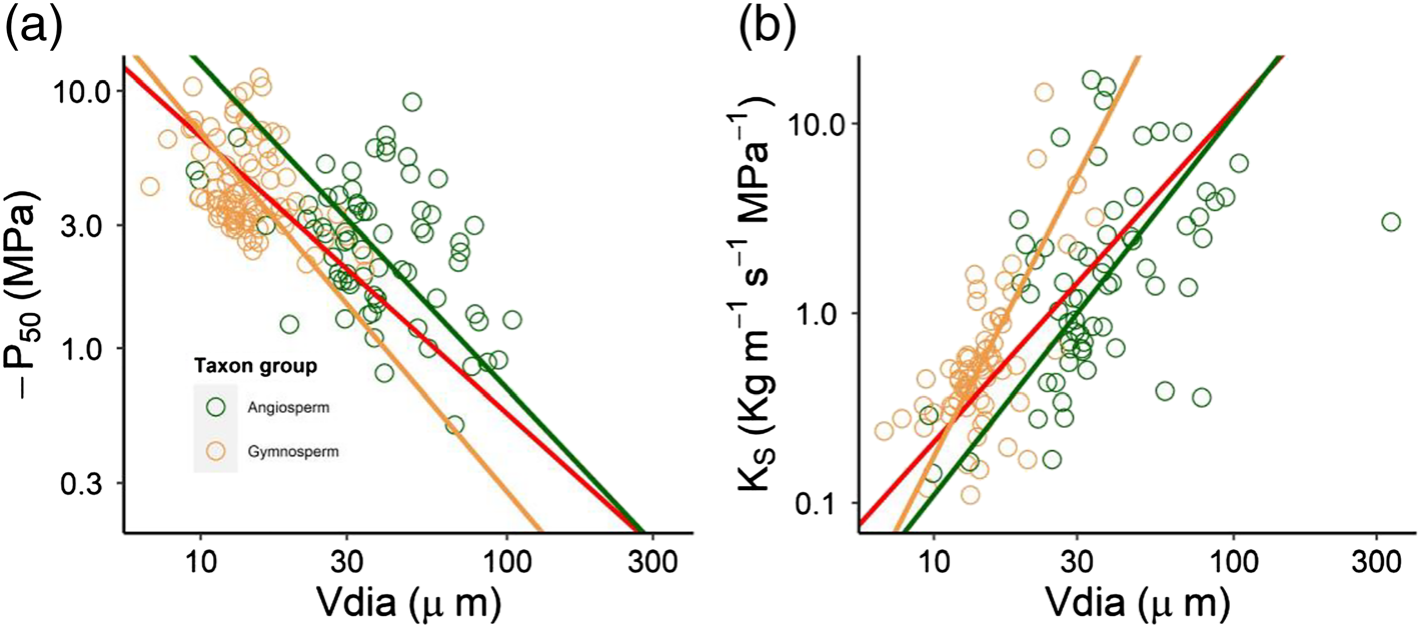
The relationships of vessel diameter with −*P*_50_ and *K*_S_. (a) *V*_dia_ is negatively coordinated with −*P*_50_ in angiosperms and gymnosperms (*n* = 159). (b) *V*_dia_ is positively coordinated with *K*_S_ in angiosperms and gymnosperms (*n* = 132). *V*_dia_, vessel diameter; *K*_S_, xylem hydraulic conductivity; −*P*_50_, xylem water potential at the loss of 50% maximum *K*_S_. Green and yellow circles represent angiosperms and gymnosperms, respectively. Red lines represent the curved line that pooled all angiosperms and gymnosperms points. All the curved lines are at a significant level (*P* < .05). The model parameters are reported in Table S2.

The results showed that *V*_dia_ was positively coordinated with MAT, MAP, and PET (Figure S3). The similar relationships between *V*_dia_ and environmental factors were both significant in angiosperms and gymnosperms (Figure 4a–d). The results showed that *V*_dia_ was negatively coordinated with −*P*_50_ and positively coordinated with *K*_S_ (Figure 5). The relationships of environmental factors with *K*_S_ and *P*_50_ showed that −*P*_50_ was negatively coordinated with MAT, MAP, and PET; on the contrary, *K*_S_ was coordinated with MAT, MAP, and PET, which implies that environmental pressures showed strong selecting power on hydraulic performance (hydraulic efficiency and safety). The relationship of −*P*_50_ with *K*_S_ revealed that the trade‐off was obviously between hydraulic efficiency and safety (Figure S4).

## 4 DISCUSSION

In the present study, we found that the limiting effect of GS on *V*_dia_ could be shifted by environmental pressures. Under the similar environmental conditions, the positive relationship was found between GS and *V*_dia_. Once the environmental conditions changed, the relationships between GS and *V*_dia_ became uncertain, in which, the relationships still keep positive or even changed negatively. *V*_dia_ as the fundamental anatomic structure of *K*_S_ and *P*_50_ was strongly coordinated with *K*_S_ and −*P*_50_. The strong environmental selection pressure on *K*_S_ and *P*_50_ explained the concerted regulation of *V*_dia_ by environmental factors. The shifted effect of environmental factors on cell size limitation by GS implies the strong plasticity of seed plants under the changed climates.

According to the widely accepted GS eco‐physiological consequences that GS could limit cell size (Beaulieu et al., 2008; Commoner, 1964; von Sachs, 1893), the similar limiting effect of GS on vessel cell size should also be observed. As expected, we indeed found that GS was positively coordinated with *V*_dia_ of the species sampled from same sites. The limiting effect of GS on cell size, here is *V*_dia_, could be explained by the “selfish DNA hypothesis”. The theory holds that the self‐replicating genes with no phenotypic expression are favored by natural selection at the genome level (Doolittle & Sapienza, 1980; Orgel & Crick, 1980). In this study, the positive relationship between GS and *V*_dia_ under the same environmental conditions is consistent with the “selfish DNA hypothesis.” In contrast, our results showed that the relationships between GS and *V*_dia_ became uncertain across different environmental conditions. It implies that the limiting effect of GS on *V*_dia_ is only general under the similar environmental conditions and could be possibly shifted by environmental pressures. Indeed, we found that the plants from each family experienced strong environmental pressures (Figure 3). The strong external environmental pressures shifted the limiting effect of the GS on *V*_dia_. Thereby, in the case of GS as the only driving force, the relationships between GS and *V*_dia_ would keep positive. In the case of the driving force of GS and environmental factors on *V*_dia_ in different directions (for instance, across different environmental conditions), the relationships between GS and *V*_dia_ would become uncertain, which still keep positive or even changed negatively (Figure 1).

Plant hydraulic conductivity and safety are closely coordinated with *V*_dia_. Our results showed positive relationships between *V*_dia_ and MAT, MAP, and PET. It implies that the vessel diameter is prone to evolve bigger in adaption to the warmer and moister climates. Previous studies have reported that great *V*_dia_ is associated to great *K*_S_ and low −*P*_50_ because of *V*_dia_ as the anatomic structure of *K*_S_ and *P*_50_ (Lens et al., 2011; Scoffoni et al., 2016; Tataranni et al., 2015). In the present study, we also found the strong positive relationship between *V*_dia_ and *K*_S_ and the strong negative relationship between *V*_dia_ and −*P*_50_ (Figure 5). *K*_S_ and *P*_50_ are two key indicators representing plants' hydraulic efficiency and safety, respectively, and they are often at a trade‐off (Fan et al., 2017; Grossiord et al., 2020; van der Sande et al., 2019) (Figure S4). Species that evolved specific *K*_S_ and *P*_50_ in different climate conditions are thought of as adaption or plasticity (Choat et al., 2018; Liu et al., 2019). In warmer and moister climates, great *K*_S_ and low −*P*_50_ evolved because of sufficient water supply and low hydraulic failure risk. Conversely, low *K*_S_ and great −*P*_50_ evolved in the arid and relatively cold environmental conditions because of the insufficient water supply and high hydraulic failure risk (Choat et al., 2018; Grossiord et al., 2020; Lens et al., 2011) (Figure S3). The environment selection of *K*_S_ and −*P*_50_ is ultimately reflected by anatomic structure (Pfautsch, 2016; Scoffoni et al., 2016). Thereby, the concerted regulation of environmental factors on *V*_dia_ could be explained by the environmental selection on hydraulic performance.

The shift of the limiting effect of GS on *V*_dia_ by environmental pressures indicates the combinative regulation of plant traits by intrinsic and environmental factors. GS is the intrinsic trait regulating cell size where the large GS would contribute to big *V*_dia_; meanwhile, the large GS is also the burden depressing cell fitness and is accompanied by slow metabolism and growth rate (Simonin & Roddy, 2018; Vinogradov, 2003). It is thus possible that seed plants with large GS show less environmental plasticity compared with plants with small GS. Indeed, we found that the relationship between GS and *V*_dia_ was overall positive in gymnosperms consistent with the theory that GS limits cell size; however, we found that the limiting effect of GS on *V*_dia_ was completely reversed in angiosperms, regardless of insignificance (Figure 4a). It implies the great plasticity of angiosperms (small GS) than gymnosperms (large GS), which possibly explains the great success of angiosperms relative to gymnosperms in the terrestrial ecosystem, particularly in wet–hot climates (Lamy et al., 2014). Thereby, the evolution of genome downsizing and the plasticity to changed environment together regulate plant growth, survival, and reproduction through deep time. Combined together, the findings in this study using xylem vessel cell diameter provided the new evidence of the plant performance regulated by the intrinsic and environmental drivers.

In conclusion, we provided clear evidence that the limiting effect of GS on *V*_dia_ could be shifted by environmental factors in the present study. The regulation of *V*_dia_ by environmental factors could be explained by the environmental selection on hydraulic performance. The findings provided critical insight and deepened our understanding of the combinative regulating mechanism on plant performance by intrinsic and environmental factors. The comprehensive cell size driving mechanism would initiate wide researcher interests in the relevant fields and narrow the gap between molecular and macroecology research.

## CONFLICT OF INTEREST

The authors do not have any conflict of interest to declare.

## AUTHOR CONTRIBUTIONS

Wenzhi Zhao and Xiangyan Feng provided the idea and designed the experiment. Xiangyan Feng, Linfei Zhong, Hai Zhou, Jingwen Bi, Huma Batool, and Xintan Zhang conducted the investigation, processed data, and prepared the figures. Xiangyan Feng and Wenzhi Zhao wrote and reviewed the manuscript.

## Supporting information


**Figure S1.** The extended examples of the limiting effect of genome size on vessel diameter was shifted by environmental factors. (a), (b) In the same sampling sites, the relationship between GS and *V*_dia_ is positive in the two angiosperms families (n = 2, n = 2, respectively). (c), (d) The relationship between GS and *V*_dia_ became uncertain when analyzed across sites with different environment conditions in the same families (n = 4, n = 17, respectively). (c) The relationships keeps positive or (d) changed negatively. Each circle represents one species. The circles with same color represent the species in the family sampled from the same site. In (a) and (b), the sites number and potential evapotranspiration are showed in labels. In (c) and (d), the potential evapotranspiration are represented by circle size. Different colors represent different sampling sites as the legend showed. The colored circle without site information are labeled by blank space in (d). GS, genome size; *V*_dia_, vessel diameter, PET, potential evapotranspiration.
**Figure S2.** The relationship between genome size and vessel diameter becomes uncertain across different environmental conditions. (a), (b), (c), (d), (e), (f), (g), (h) The relationship between GS and *V*_dia_ became uncertain when analyzed across sites with different environment conditions in the same families (n = 5, n = 2, n = 6, n = 2, n = 3, n = 2, n = 3, n = 2, respectively). (b), (d), (f) The relationships keeps positive or (a), (c), (e), (g), (h) changed negatively. Each circle represents one species. The sites potential evapotranspiration are represented by circle size. GS, genome size; *V*_dia_, vessel diameter, PET, potential evapotranspiration.
**Figure S3.** The relationships of ‐*P*_50_ and *K*_S_ with environmental pressures. (a), (b), (c) ‐*P*_50_ is negatively coordinated with MAT, MAP, and PET (n = 380, n = 380, n = 380, respectively). (e), (d), (f) *K*_S_ is positively coordinated with MAT, MAP, and PET (n = 252, n = 252, n = 252, respectively). *K*_S_, xylem hydraulic conductivity; ‐*P*_50_, xylem water potential at the loss of 50% maximum *K*_S_; MAT, mean annual temperature; MAP, mean annual precipitation; PET, potential evapotranspiration. Blue and yellow circles represent angiosperms and gymnosperms, respectively. All the curved lines is at the significant level (*P* < .05). The model parameters are reported in the Table S2.
**Figure S4.** The relationship between hydraulic conductivity and safety. *K*_S_ is negatively coordinated with ‐*P*_50_ (n = 202). *K*_S_, xylem hydraulic conductivity; ‐*P*_50_, xylem water potential at the loss of 50% maximum *K*_S_. Blue and yellow circles represent angiosperms and gymnosperms, respectively. The curved lines is at the significant level (*P* < .05). The model parameters are reported in the Table S2.

**Table S1.** The dataset compiled.

**Table S2.** The Model parameters of the relationships among varibles.
