# Supplementary material for: The limiting effect of genome size on xylem vessel diameter is shifted by environmental pressures in seed plants
Source: Plant Direct. 2022 Dec 14;6(12):e471. doi: 10.1002/pld3.471 (PMC9751660; doi:10.1002/pld3.471)
Supplement: Supplementary file 1 — Figure S1. The extended examples of the limiting effect of genome size on vessel diameter was shifted by environmental factors. (a), (b) In the same sampling sites, the relationship between GS and V dia is positive in the two angiosperms families (n = 2, n = 2, respectively). (c), (d) The relationship between GS and V dia became uncertain when analyzed across sites with different environment conditions in the same families (n = 4, n = 17, respectively). (c) The relationships keeps positive or (d) changed negatively. Each circle represents one species. The circles with same color represent the species in the family sampled from the same site. In (a) and (b), the sites number and potential evapotranspiration are showed in labels. In (c) and (d), the potential evapotranspiration are represented by circle size. Different colors represent different sampling sites as the legend showed. The colored circle without site information are labeled by blank space in (d). GS, genome size; V dia, vessel diameter, PET, potential evapotranspiration. Figure S2. The relationship between genome size and vessel diameter becomes uncertain across different environmental conditions. (a), (b), (c), (d), (e), (f), (g), (h) The relationship between GS and V dia became uncertain when analyzed across sites with different environment conditions in the same families (n = 5, n = 2, n = 6, n = 2, n = 3, n = 2, n = 3, n = 2, respectively). (b), (d), (f) The relationships keeps positive or (a), (c), (e), (g), (h) changed negatively. Each circle represents one species. The sites potential evapotranspiration are represented by circle size. GS, genome size; V dia, vessel diameter, PET, potential evapotranspiration. Figure S3. The relationships of ‐P 50 and K S with environmental pressures. (a), (b), (c) ‐P 50 is negatively coordinated with MAT, MAP, and PET (n = 380, n = 380, n = 380, respectively). (e), (d), (f) K S is positively coordinated with MAT, MAP, and PET (n = 252, n = 252, n = 252, respectivel [file PLD3-6-e471-s003.docx]

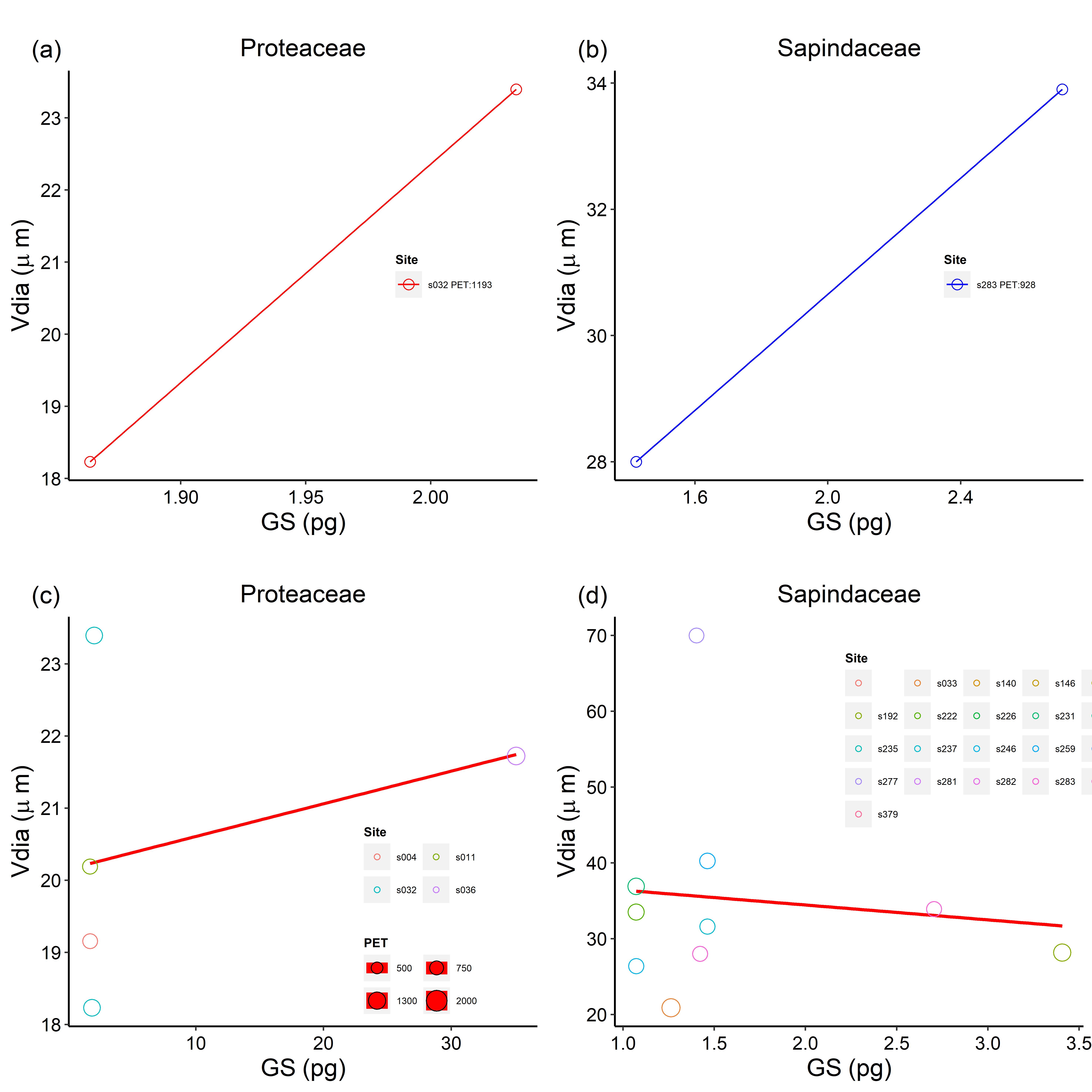


Fig. S1 The extended examples of the limiting effect of genome size on vessel diameter was shifted by environmental factors. (a), (b) In the same sampling sites, the relationship between GS and *V*_dia_ is positive in the two angiosperms families (n=2, n=2, respectively). (c), (d) The relationship between GS and *V*_dia_ became uncertain when analyzed across sites with different environment conditions in the same families (n=4, n=17, respectively). (c) The relationships keeps positive or (d) changed negatively. Each circle represents one species. The circles with same color represent the species in the family sampled from the same site. In (a) and (b), the sites number and potential evapotranspiration are showed in labels. In (c) and (d), the potential evapotranspiration are represented by circle size. Different colors represent different sampling sites as the legend showed. The colored circle without site information are labeled by blank space in (d). GS, genome size; *V*_dia_, vessel diameter, PET, potential evapotranspiration.


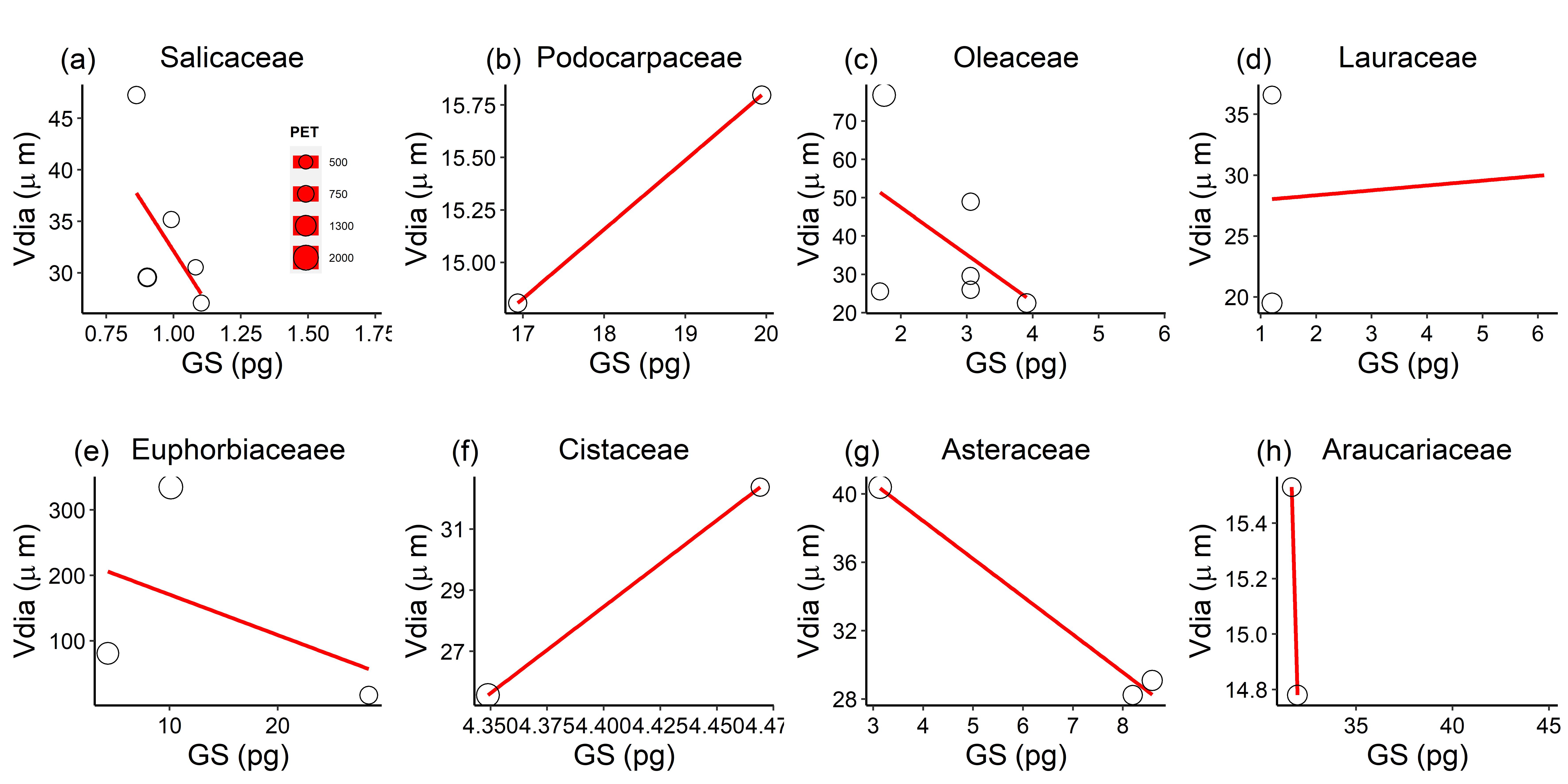


Fig. S2 The relationship between genome size and vessel diameter becomes uncertain across different environmental conditions. (a), (b), (c), (d), (e), (f), (g), (h) The relationship between GS and *V*_dia_ became uncertain when analyzed across sites with different environment conditions in the same families (n=5, n=2, n=6, n=2, n=3, n=2, n=3, n=2, respectively). (b), (d), (f) The relationships keeps positive or (a), (c), (e), (g), (h) changed negatively. Each circle represents one species. The sites potential evapotranspiration are represented by circle size. GS, genome size; *V*_dia_, vessel diameter, PET, potential evapotranspiration.


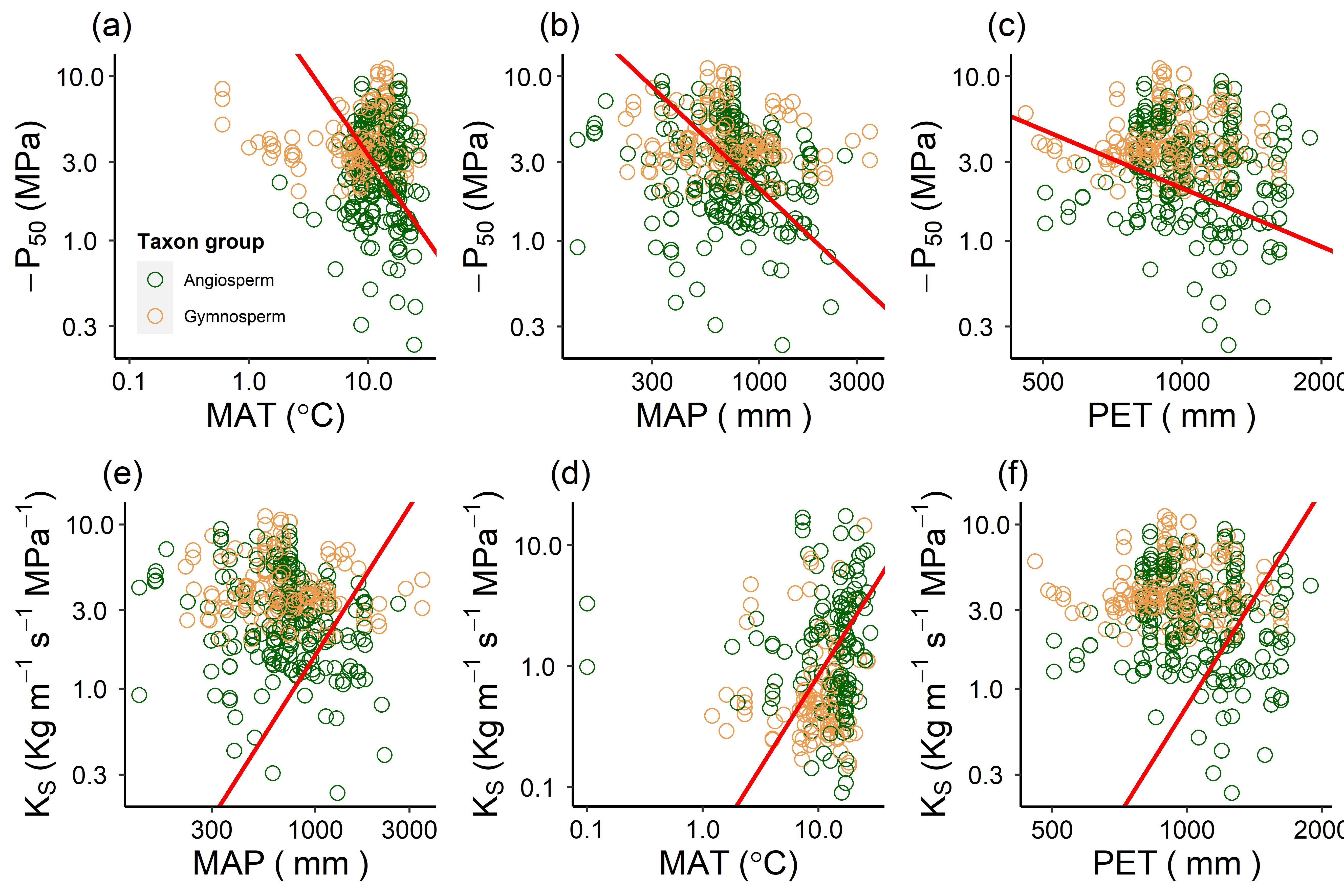


Fig. S3 The relationships of -*P*_50_ and *K*_S_ with environmental pressures. (a), (b), (c) -*P*_50_ is negatively coordinated with MAT, MAP, and PET (n=380, n=380, n=380, respectively). (e), (d), (f) *K*_S_ is positively coordinated with MAT, MAP, and PET (n=252, n=252, n=252, respectively). *K*_S_, xylem hydraulic conductivity; -*P*_50_, xylem water potential at the loss of 50% maximum *K*_S_; MAT, mean annual temperature; MAP, mean annual precipitation; PET, potential evapotranspiration. Blue and yellow circles represent angiosperms and gymnosperms, respectively. All the curved lines is at the significant level (*P*<0.05). The model parameters are reported in the Table S2.


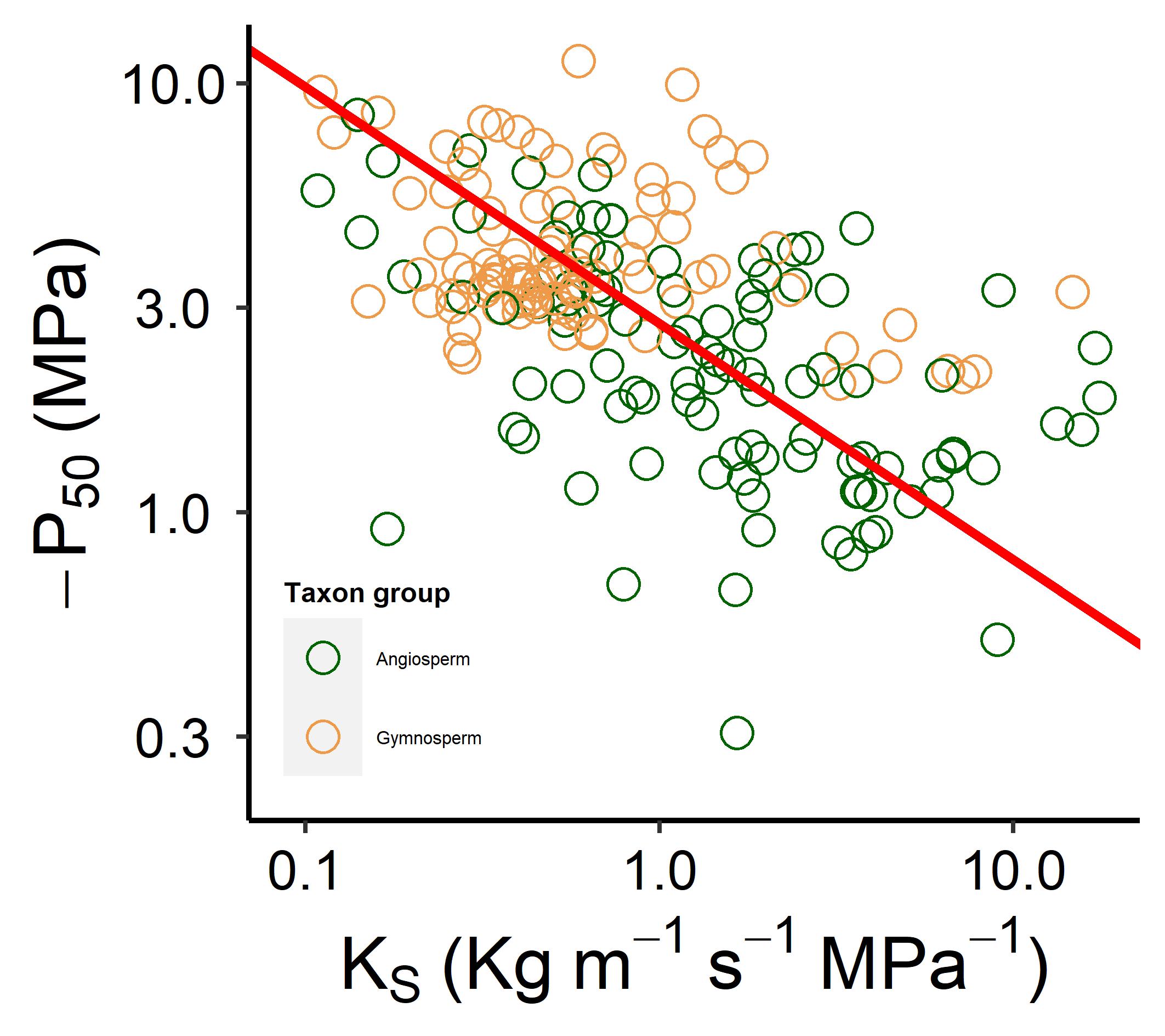


Fig. S4 The relationship between hydraulic conductivity and safety. *K*_S_ is negatively coordinated with -*P*_50_ (n=202). *K*_S_, xylem hydraulic conductivity; -*P*_50_, xylem water potential at the loss of 50% maximum *K*_S_. Blue and yellow circles represent angiosperms and gymnosperms, respectively. The curved lines is at the significant level (*P*<0.05). The model parameters are reported in the Table S2.
