# Supplementary material for: The limiting effect of genome size on xylem vessel diameter is shifted by environmental pressures in seed plants
Source: Plant Direct. 2022 Dec 14;6(12):e471. doi: 10.1002/pld3.471 (PMC9751660; doi:10.1002/pld3.471)
Supplement: Supplementary file 3 — Table S2. The Model parameters of the relationships among varibles. [file PLD3-6-e471-s002.docx]

Table S2. The Model parameters of the relationships among varibles.

| **Figures** | **Group** | **y** | **x** | **n** | ***R*^2^** | ***P*** | **Slope** | **Intercept** |
| --- | --- | --- | --- | --- | --- | --- | --- | --- |
| Fig. 3a | A/G | Vdia | GS | 185 | 0.47 | 0.0001 | -0.43 | 1.76 |
| Fig. 3a | A | Vdia | GS | 95 | 0.004 | 0.54 | -0.7 | 1.8 |
| Fig. 3a | G | Vdia | GS | 90 | 0.001 | 0.73 | 0.81 | -0.09 |
| Fig. 3b | A/G | Vdia | MAT | 178 | 0.11 | 0.0001 | 1.14 | 0.19 |
| Fig. 3b | A | Vdia | MAT | 91 | 0.08 | 0.007 | 1.36 | 0.06 |
| Fig. 3b | G | Vdia | MAT | 87 | 0.002 | 0.72 | -0.45 | 1.58 |
| Fig. 3c | A/G | Vdia | MAP | 178 | 0.006 | 0.31 | 1.22 | -2.09 |
| Fig. 3c | A | Vdia | MAP | 91 | 0.06 | 0.02 | 0.92 | -1.01 |
| Fig. 3c | G | Vdia | MAP | 87 | 0.002 | 0.7 | -0.66 | 3.01 |
| Fig. 3d | A/G | Vdia | PET | 178 | 0.2 | 0.0001 | 2.36 | -5.71 |
| Fig. 3d | A | Vdia | PET | 91 | 0.15 | 0.0001 | 2.06 | -4.71 |
| Fig. 3d | G | Vdia | PET | 87 | 0.01 | 0.36 | -1.23 | 4.81 |
| Fig. 4a | A/G | P50 | Vdia | 159 | 0.34 | 0.0001 | -0.93 | 1.76 |
| Fig. 4a | A | P50 | Vdia | 71 | 0.13 | 0.002 | -1.26 | 2.36 |
| Fig. 4a | G | P50 | Vdia | 88 | 0.14 | 0.0003 | -1.39 | 2.23 |
| Fig. 4b | A/G | Ks | Vdia | 132 | 0.4 | 0.0001 | 1.75 | -2.43 |
| Fig. 4b | A | Ks | Vdia | 65 | 0.22 | 0.0001 | 2 | -2.96 |
| Fig. 4b | G | Ks | Vdia | 67 | 0.4 | 0.0001 | 3.09 | -3.84 |
| Fig. S3a | A/G | P50 | MAT | 380 | 0.01 | 0.04 | -1.04 | 1.56 |
| Fig. S3b | A/G | P50 | MAP | 380 | 0.03 | 0.0004 | -1.18 | 3.86 |
| Fig. S3c | A/G | P50 | PET | 380 | 0.03 | 0.001 | -2.5 | 7.97 |
| Fig. S3d | A/G | Ks | MAT | 252 | 0.02 | 0.04 | 1.53 | -1.6 |
| Fig. S3e | A/G | Ks | MAP | 252 | 0.09 | 0.0001 | 1.9 | -5.5 |
| Fig. S3f | A/G | Ks | PET | 252 | 0.05 | 0.0004 | 4.38 | -13.23 |
| Fig. S4 | A/G | Ks | P50 | 202 | 0.29 | 0.0001 | -0.55 | 0.44 |

A, angiosperms; G, gymnosperms; Vdia, vessel diameter; Ks, xylem hydraulic conductivity; P50, xylem water potential at the loss of 50% maximum Ks; MAT, mean annual temperature; MAP, mean annual precipitation; PET, potential evapotranspiration.
